# Stabilization and Anticancer Enhancing Activity of the Peptide Nisin by Cyclodextrin-Based Nanosponges against Colon and Breast Cancer Cells

**DOI:** 10.3390/polym14030594

**Published:** 2022-02-01

**Authors:** Yousef Khazaei Monfared, Mohammad Mahmoudian, Claudio Cecone, Fabrizio Caldera, Parvin Zakeri-Milani, Adrián Matencio, Francesco Trotta

**Affiliations:** 1 Dipartimento Di Chimica, Università di Torino, Via P. Giuria 7, 10125 Torino, Italy; yousef.khazaeimonfared@unito.it (Y.K.M.); claudio.cecone@unito.it (C.C.); fabrizio.caldera@unito.it (F.C.); 2 Faculty of Pharmacy, Tabriz University of Medical Sciences, Tabriz 5166414766, Iran; mahmoudian.m@tbzmed.ac.ir; 3 Liver and Gastrointestinal Diseases Research Centre and Faculty of Pharmacy, Tabriz University of Medical Sciences, Tabriz 5166414766, Iran

**Keywords:** β-cyclodextrin-based nanosponges, Nisin-Z stability, anti-cancer, MCF-7, HT-29

## Abstract

The great variability of cancer types demands novel drugs with broad spectrum, this is the case of Nisin, a polycyclic antibacterial peptide that recently has been considered for prevention of cancer cells growth. As an accepted food additive, this drug would be very useful for intestinal cancers, but the peptide nature would make easier its degradation by digestion procedures. For that reason, the aim of present study to investigate the protective effect of two different β-cyclodextrin-based nanosponges (carbonyl diimidazole and pyromellitic dianhydride) and their anti-cancer enhancement effect of Nisin-Z encapsulated with against colon cancer cells (HT-29). To extend its possible use, a comparison with breast (MCF-7) cancer cell was carried out. The physicochemical properties, loading efficiency, and release kinetics of Nisin complex with nanosponges were studied. Then, tricin-SDS-PAGE electrophoresis was used to understand the effect of NSs on stability of Nisin-Z in the presence of gastric peptidase pepsin. In addition, the cytotoxicity and cell membrane damage of Nisin Z were evaluated by using the MTT and LDH assay, which was complemented via Annexin-V/ Propidium Iodide (PI) by using flowcytometry. CD-NS are able to complex Nisin-Z with an encapsulation efficiency around 90%. A protective effect of Nisin-Z complexed with CD-NSs was observed in presence of pepsin. An increase in the percentage of apoptotic cells was observed when the cancer cells were exposed to Nisin Z complexed with nanosponges. Interestingly, Nisin Z free and loaded on PMDA/CDI-NSs is more selectively toxic towards HT-29 cells than MCF-7 cancer cells. These results indicated that nanosponges might be good candidates to protect peptides and deliver drugs against intestinal cancers.

## 1. Introduction

Currently, cancer is one of the most important diseases to get a universal therapy due to the diversity of cancer lines [1]. In particular, colon cancer caused nearly 881,000 deaths in 2018 [2]. To overcome this challenge, the research of novel broad spectrum drugs is one of the most promising alternatives. Recently, there has been reported the anti-cancer effect of food preservative and bacteriocin agent, Nisin [3,4,5], a polycyclic antibacterial peptide containing 34-amino acid with rarely amino acids, such as dehydroalanine (Dha), dehydrobutirine (Dhb), lanthionine (Lan), and methyl-lanthionine (Melan) [2]. Thermal stability and antibacterial activity of Nisin are related to these unusual amino acids [2,6,7]. Different variants of Nisin have been discovered from a range of taxonomically distinct organisms isolated from a broad range of environments [6]. The differences among Nisin-Z and Nisin A, the first discovered natural Nisin, is attributed to the present histidine and asparagine amino acids in the residue position 27, respectively, which led to enhance the diffusion of Nisin Z [7]. Increased solubility and stability of Nisin in acidic medium as well as its stability in autoclave, at 121 °C for 15 min and pH 2–3 without denaturation, has been reported [8]. While Nisin mainly has been used in the food industry as a food protective against bacterial growth through disrupting the integrity and formation of the short-lived pore inside the bacterial cells, leading to a change in membrane potential [9], it has only recently been considered for prevention of cancer cells growth: in the last few years, extensive research has confirmed that the cytotoxicity and antitumor effects of Nisin are mediated through the activation of preferential apoptosis, cell cycle arrest, and reduced cell proliferation [3,10,11,12,13]. The cationic amino acids moieties of the Nisin interact with negatively charged phospholipid heads from the cell membrane of the molecule, while the hydrophobic portion of Nisin interacts with the nucleus of the membrane core [14].

Protein and peptide-based drugs have shown great biological activities against cancer cells because of their high biocompatibility, high specificity, and low toxicity as new therapeutically agents with a broad-spectrum of anti-cancer activities to inhibit tumor growth [15,16,17]. Whereas, the main limitation in regard to these agents is low stability in-vivo due to enzymatic cleavage by peptidases, therefore, some delivery approaches were implied to improve plasma half-life of the peptide drugs [16,18,19]. On the other hand, the delivery of effective and safe therapeutic agents to human cancer cells has been studied by various methods [20,21,22,23,24,25]. Among others, polymer science is used to design such drug delivery systems against cancer cells. As a potential polymer system, cyclodextrin-based (CD-NS) or cross-linked cyclodextrins nanosponges have often been used in drug delivery systems to deliver various anticancer drugs due to their low cost and high biocompatibility [26]. Cyclodextrins (CD), the monomer unit, is a cyclic oligosaccharides containing glucopyranoside monomeric units linked via α-(1,4)-glycosidic bonds with the central lipophilic cavity and outer hydrophilic surface [27]. These monomers can be linked to different type of cross-linkers such as carbonyl diimidazole (CDI) and pyromellitic dianhydride (PMDA) to make a highly cross-linked system [28,29]. Previous reports have shown that the main important properties of CDNSs in drug delivery are related to their high capacity to encapsulate different molecules with solubilization improvement power for poorly water-soluble drugs and its properties [30] protect drugs from physicochemical degradation [31] and extend the drug release [32] through intravenous, oral, and pulmonary pathways because they are biodegradable and safe [33]. 

Nisin-Z as anti-tumor agent can be decomposed by proteases in the digestive system, while it is unstable at physiological pH. Consequently, these issues are the main concerns to use this peptide in clinical practice [34,35,36]. Therefore, the aim of the present study to investigate the enhancement of stability and anti-cancer effect of Nisin Z encapsulated with two different CDNSs (PMDA-NSs and CDI-NSs) against colon and breast cancer cells in vitro.

## 2. Materials and Methods

### 2.1. Materials

Nisin Z (2.5% *w*/*w*, N5764), Pepsin (P0525000), MTT (M2003), and Cytotoxicity Detection Kit ^PLUS^ (LDH) were obtained from Sigma Aldrich (Milan, Italy). FITC Annexin-V apoptosis kit I (BD Pharmigen™, BD Biosciences, and San Jose, CA, USA, 556547) and fetal bovine serum (FBS) were purchased of Cegrogen-Biotech (Germany, A0100-3010). Pen-Strep (10,000 Unit/mL penicillin and 10,000 unit/mL streptomycin) and 0.25% trypsin-EDTA were purchased from Bioidea (Tehran, Iran). RPMI was prepared from GIBCO Laboratories (Grand Island, NY, USA, 11530586). Coumarin-6 and Oxaliplatin were kindly gifted by Professor Valizadeh’s laboratory (TUOMS, Tabriz, Iran). All other chemicals were of analytical grade.

### 2.2. Synthesis and Characterization of β-Cyclodextrin-Nanosponges 

There was prepared two kinds of β-CDNSs by using molar proportions of β-CD with CDI and PMDA (1:4) as previously reported, briefly, 20 mL of DMSO solution containing 6.1 g of β-CD, 2.34 g of PMDA and 0.7 mL of triethylamine were reacted for 3 h at room temperature. The product was Soxhlet, which was extracted with acetone for 24 h. Purified PMDA-NS were dried at 60 °C for 24 h and stored at room temperature until further use. CDI-NSs formulation was made based on previous reports, β-CD (5 g) was dissolved in N, N-dimethylformamide (30 mL), and CDI (2.852 g) was added into it. The reaction was performed at 90 °C for 3 h to obtain a solid mass, which was crushed and washed with water and then washed with acetone to remove unreacted monomers. [37,38]. Furthermore, nanosponges were purified in ethanol by Soxhlet extraction for 24 h. The solid obtained was stored in a desiccator at 25 °C for further usage. 

#### 2.2.1. Preparation of Nisin-Loaded β-CDNSs and Coumarin-6 CDNSs

Nisin Z loaded CDNSs 1:4 were prepared by freeze-drying method. Briefly, a weighed amount of CDNSs 1:4 was suspended in 20 mL of Milli Q water under stirring, then the calculated amount of Nisin in a weight ratio of 1:4 (Drug: CDNS) was added. The mixture was sonicated for 15 min and kept for 24 h under stirring. To remove uncomplexed drug suspensions, it was centrifuged at 2000 rpm for 15 min. The precipitated was dried in an oven at 60 °C in vacuumed pressure. The dried powder was grinded and stored in a desiccator. In addition, CD-NSs with fluorescent dyes were built in a similar way by adding the fluorescent marker 6-coumarin (0.1 mg/mL) to the aqueous nanosuspension of CDNSs (10 mg/mL) in the dark condition.

#### 2.2.2. Determination of Nisin Content in β-CDNSs

To break the complex, a determined amount of Nisin-CDNSs complexes were dispersed in Milli Q water and sonicated for 10 min. The protein concentration in the solution was determined by the Bradford reagent, which is due to the formation of a complex between the dye, Brilliant Blue G, and the proteins in the solution. The protein–dye complex has a maximum absorption at 595 nm. Bradford reagent was used to detect low concentrations of protein molecules in solutions due to its high sensitivity [39]. The encapsulation efficiency (EE) and Drug Loading (%DL) of the drug was calculated according to the following formulas:

Drug Loading (%DL) = Total drug (mg) − Free drug (mg)/ Total amount of NPs (mg) × 100(1)

Encapsulation Efficiency (% EE) = Total drug (mg) − Free drug (mg)/ Total drug (mg) × 100(2)

#### 2.2.3. Size Characterization of Synthesized β-CDNSs

The 90 plus particle sizer (Malvern Instruments, Malvern, UK) was used at the fixed angel of 90° and 25.0 °C to measure the size and polydispersity index(PdI) of β-CD and CDNSs. A total of 5 mg of samples were suspended in distilled water (5 mL) and sonicated for 15 min to avoid the presence of aggregates. The same instrument was used to measure the zeta potential. 

#### 2.2.4. Differential Scanning Calorimetry Analysis

Differential scanning calorimetry (DSC) studies were done by using a PerkinElmer, Jade instrument on plain Nisin, CDNS, encapsulated, and physical mixture of the mentioned components. An empty pan was used as a reference standard. Samples heated in the 30–300 °C temperature range and the rate of 10 °C/min.

#### 2.2.5. In Vitro Studies of Nisin Release

Nisin Z release profile was evaluated by suspending 2 mL of a 25 mg/mL solution of Nisin Z and CDNSs suspension, including the same quantity of entrapped Nisin Z into 20 mL of PBS solution (0.137 M NaCl, 0.0027 M KCl, 0.01 M Na2HPO4, and 0.0018 M KH2PO, pH 7.4) for 48 h at 37 °C. The suspensions were stirred and then sonicated for 5 min and kept at incubator (37 °C) under shaking at 150 rpm. At the appointed times, 0.5 mL of sample were collected from the suspension and transferred into the Amicon^®^ tube (Ultra-30 kDa molecular weight cut-off membrane, Millipore, Germany) and centrifuged at 3500× *g* for 10 min [10]. Bradford’s method was implied for quantifying the amount of Nisin in the buffer, as earlier explained. All experiments were done in triplicate.

### 2.3. Screening of Biological Activity

#### 2.3.1. Cell Culturing Conditions

HT-29 (Passage Number: 4) and MCF-7 (Passage Number: 8) cells were obtained from Pasture Institute National Cell Bank of Tehran, Iran. The cells were cultivated in RPMI 1640 supplemented with 10% FBS and 1% Penicillin/Streptomycin solution. The flask was incubated in a humidified incubator at 37  °C containing 5% CO_2_. The media was exchanged every other day, and the cells were detached and harvested after trypsinization at 80~90% confluence in each passage. 

#### 2.3.2. Cellular Uptake Study 

Cellular uptake was performed to evaluate the effectiveness of CDNS internalization within cancer cells. Nano-carrier was labeled by Coumarin-6 (C-6) as a fluorescent molecule model. The Amicon^®^ tube (Ultra-30 kDa molecular weight cut-off membrane, Millipore, Germany) was used to purify free C-6 from nanoparticles. The cellular uptake of coumarin-6-encapsulated with PMDA-NSs and CDI-NSs in cancer cells was qualitatively and quantitatively studied. The cancer cells were seeded in 6-well plate at a density of 5 × 10^5^ cell/well, incubated overnight, and then treated with coumarin-6-loaded CDNSs for 4 h, thereafter, the cells were observed by Cytation 5 Cell Imaging Multi-Mode Reader (San Jose, CA, USA, Gen5™ software). Quantitative cellular uptake of CDNSs in cancer cells were determined using flow cytometry. The cells at a density of 200 × 10^4^ cell/well were seeded in 12-well plate and incubated for 24 h to obtain an 80% confluency. The cells were treated with CDNS containing C-6 for 4 h and kept in an incubator with 5% CO_2_ at 37 °C. After that, the cells trypsinized and washed with PBS to analyze the uptake potential of CDNSs with Fluorescence-activated Cell Sorting (FACS) caliber flow cytometer (BD Pharmigen™, BD Biosciences, and San Jose, CA, USA) based on the fluorescent intensity.

#### 2.3.3. In Vitro Cytotoxicity Assays

For toxicity assays, cells were seeded at a density of 25,000 cells per well in 96-well plates. A 250, 125, and 62 µg/mL solution of Nisin Z and CDNSs suspension containing the same amount of entrapped Nisin Z were freshly prepared prior to each experiment. All experiments were performed in serum-free RPMI1640 medium. MTT and LDH assays were used to determine the cytotoxicity of the Nisin free and in complexes. Finally, the flow cytometric assay was tested to define the apoptosis and necrosis of cancer cells. All experiments were at least performed in duplicate and repeated independently.

#### 2.3.4. MTT (3-(4, 5-dimethylthiazol-2-yl) 2, 5-diphenyl Tetrazolium Bromide) Assay

The 3-(4, 5-dimethylthiazol-2-yl) 2, 5-diphenyl tetrazolium bromide (MTT) assay was used to determine in vitro cell viability. HT-29 and MCF-7 cells were seeded in 96-well plates and incubated until cells were 80% confluent, followed by exposure to Nisin Z (250, 125, and 62 µg/mL), CDNSs suspension containing the same amount of entrapped Nisin Z and CDNSs free of drug for 24 h. Vehicle control groups were also included. Following exposure, growth medium was removed, cells carefully rinsed with 1X phosphate buffered saline (PBS), and 100 µL fresh serum-free medium containing 0.5 mg/mL MTT solution added. Cells were then incubated for 4 h at 37 C, after which the MTT was removed and replaced with 100 µL dimethyl sulfoxide (DMSO). After 1 h of incubation at 37 °C, cell viability was determined using a micro- plate ELISA reader (Biotech, San Jose, CA, USA, Gen5™ software). Absorbance was measured at a wavelength of 560 nm and background at a wavelength of 630 nm with DMSO measured as a blank. Blank and background measurements were subtracted and cell viability is expressed as a percentage relative to the untreated control, which was set as 100% viable. The cell viability was calculated based on the optical density (OD) values of the treated and un-treated cells using the following equation:

Cell Viability %= (OD _treated_ − OD _blank_)/(OD _untreated_ − OD _Blank_) × 100 (3)

#### 2.3.5. Lactate Dehydrogenase (LDH) Assay

Cell membrane integrity was evaluated using the Plus Cytotoxicity Detection Kit (method) according to the manufacturer’s protocol with some modifications. Briefly, HT-29 and MCF-7 cells were seeded in a 96-well plate and incubated until cells were 80% confluent. Cells were thereafter exposed to Nisin Z (250, 125, and 62 µg/mL), CDNSs suspension containing the same amount of entrapped Nisin Z and CDNSs free of drug for 24 h. A total of 100 µL of freshly prepared Reaction Mixture was added into each well and incubated 10 min at 25 °C under dark. A total of 50 µL Stop Solution was then added into each well, thereafter, the plate was shaken for 10 sec for quantifying the LDH leakage. Cells were contacted to 0.01% Triton-X 100 as a positive control. The absorbance at 492 nm was measured by micro-plate ELISA reader (Biotech, USA, Gen5™ software). Results are exhibited relative to the LDH release of control negative and positive groups with 0 and 100 percentage, respectively.

#### 2.3.6. Determine the Apoptosis Assay

The FITC Annexin-V apoptosis kit I (BD Pharmigen™, BD Biosciences, and San Jose, CA, USA, 556547) has been applied to determine the apoptosis percentage. Loss of cell membrane integrity and apoptosis were defined by green fluorescence released from FITC Annexin-V, while late apoptotic or necrotic cells were identified by Propidium iodide (PI) staining. The assay was performed according to the manufacturer’s instructions, with some changes. In brief, HT-29 and MCF-7 cells were seeded in a 12-well plate and grown to 80% confluence. Cells were then exposed to 250 µg/mL concentration of Nisin Z and CDNSs suspension containing the same amount of entrapped Nisin Z. After 24 h, cells were two times washed with 400 µL of fresh PBS and suspended in 1 X binding buffer. Then, 5 μL of FITC Annexin V and 5 μL of propidium iodide (PI) were added to each sample and the cells were mixed slowly and incubated in the dark at room temperature for 20 min. After incubation, 100 µL 1 X binding buffer was added to each tube and then analyzed using the BD FACSVerse™ and BD FACSuite™ Software (BD Biosciences, San Jose, CA, USA). Oxaliplatin (OxPt) and unstaining cells were analyzed as positive and negative controls, respectively. At least 10,000 cellular events were analyzed for each sample. A logarithmic amplification scale was employed and events plotted on forward scatter (FSC), side scatter (SSC), green fluorescence (FL1), and red fluorescence (FL2). Subsequent data analysis was performed using FlowJo X (10.0.7r2) single cell analysis software (FlowJo-V10, LLC). All experiments were at least performed in triplicate and independently repeated.

#### 2.3.7. Tricine-SDS-PAGE 

Tricine-sodium dodecyl sulfate-polyacrylamide gel electrophoresis (Tricine- SDS-PAGE) was done according to a previous report (25). For sample preparation, 10 μL of a 2X gel loading buffer (0.25 M Tris-HCl, pH 6.8, 5% glycerol, 5% 2-mercaptoethanol, 3% SDS, and 0.2 mg.mL^−1^ bromophenol blue) was added to 10 μL of samples containing Nisin Z free and PMDA/CDI-NSs containing the same amount of Nisin, then, samples were heated to 95 °C for 10 min. Samples were then loaded on a tricine gel consisting of a stacking gel containing 4% acrylamide, a spacer gel containing 10% acrylamide, and a separation gel containing 16.5% acrylamide. The gel was run at 30 V for 1 h and then at 80 V for 3 h. Coomassie Blue staining (G250) was used to visualize the peptide. Spectra multicolor low range protein ladder was used. Thermo scientific spectra low range protein ladder was used, ranging from 1.7 to 40 kDa.

### 2.4. Statistical Analysis 

Statistical analysis was conducted using GraphPad Prism 8 (GraphPad Software, Inc., La Jolla, CA, USA). After Shapiro–Wilk test, data were analyzed using one- and two-way ANOVA Analysis of Variance after that the Dunnett’s multiple comparisons test was used. All the samples were analyzed in triplicates and were presented as mean ± standard deviation (SD) for *n* = 3. The level of significance was calculated by *p*-value. Statistically, *p* value < 0.05 was considered significant.

## 3. Results

### 3.1. Characterization Results

**Figure 1:**
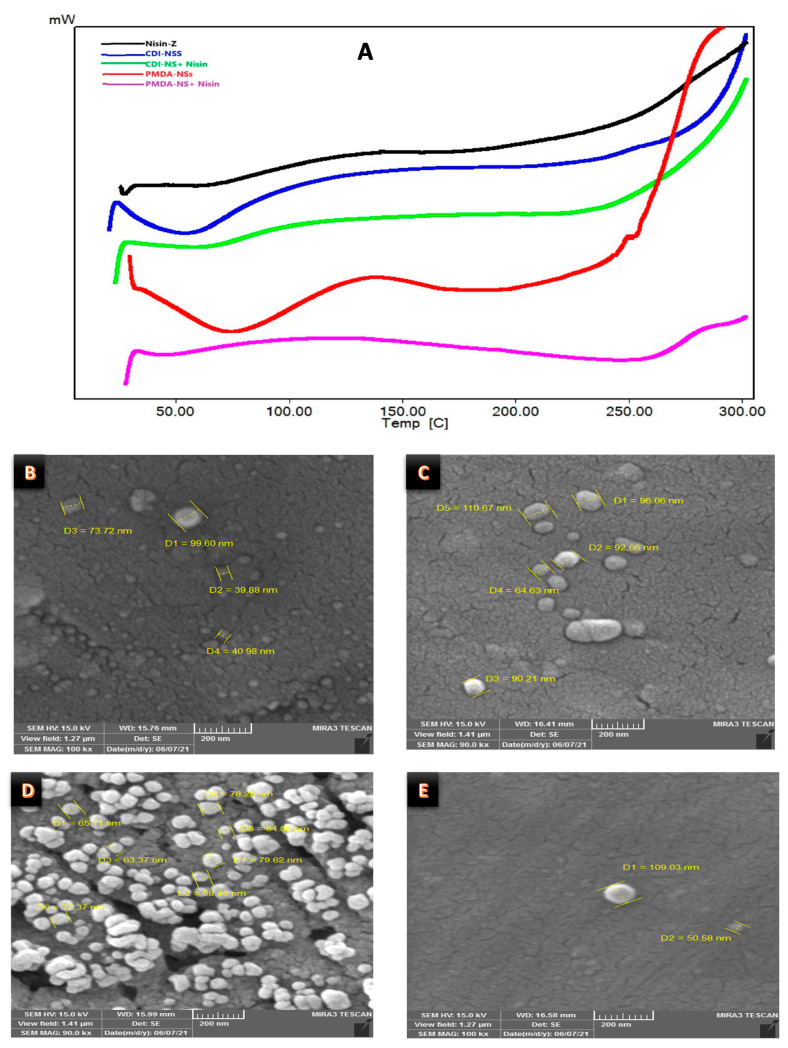
Physicochemical characterization of Nisin-Z and complexes. (**A**) DSC thermograms of Nisin, Nisin/PMDA-NSs, and Nisin/CDI-NSs, SEM images; (**B**) CDI-NSs, (**C**) Nisin/CDI-NSs, (**D**) PMDA-NSs, (**E**) Nisin/PMDA-NSs.

Firstly, a DSC was carried out to understand the complex formation. The changes in the thermogram would indicate the formation of the inclusion complex [40]. As Figure 1A shows, the variations of both CD-NS around 80–100 °C due to intrinsic water evaporation, might justify the formation of the complex, although it is not clear [40,41]. On the other hand, the SEM images showed that Nisin-Z should be included in the CD-NSs due to the morphological changes, from irregular structure to regular sphere (Figure 1B–E). However, the confirmation was found with the increase in Z-Average and the variation in the Z-potential (Table 1). Despite of the considerable PDI due to the polymeric nature of the material, the complexation of Nisin created a bigger particle than free NSs and the cationic charge of Nisin would decrease the Z potential of free NSs, as reported the experimental data and bibliography for other proteins and NSs [42]. These points justify the formation of the complex between Nisin and CD-NSs due to the intrinsic charge of Nisin.

### 3.2. Release Profile

**Figure 2:**
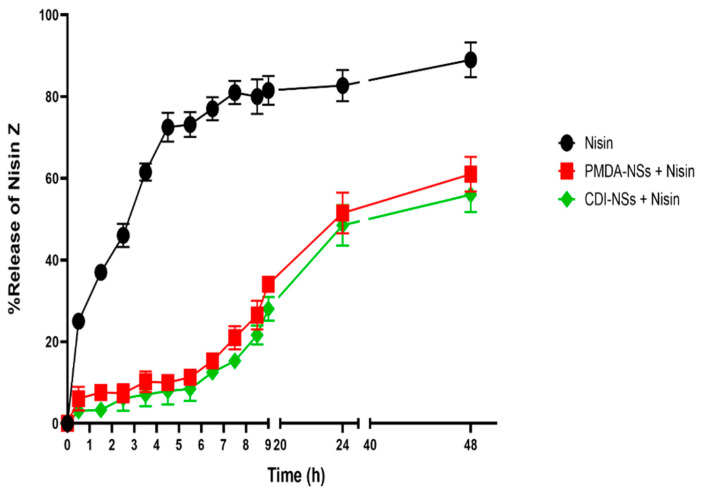
The release profile of the Nisin and Nisin-loaded-PMDA/CDI-NSs (Dissolution medium: Phosphate Buffer pH 7.4 Solution, temperature: 37 ± 0.5 °C, rotation speed: 150 rpm).

The anti-cancer activity of Nisin Z-loaded nanosponges is dependent on the release of Nisin from the polymeric matrix into the environment. Figure 2 compares the release of entrapped Nisin Z from the Nisin-loaded PMDA-NSs and CDI-NSs with free Nisin Z release. As can be seen, the in vitro release profile of NIS-NSs were slow and steady during 72 h, showing a sustained and prolonged release profile. During the first three hours, there was an initial burst effect of free Nisin release into the media. Prolonged release of Nisin in the releasing media was observed due to the increment of diffusion distance in polymer matrix. Nisin loaded nanosponges resulted in only about 35% Nisin immediate release as compared to up to 80% release during the first to fourth hour. This outcome confirmed the entrapment of Nisin inside the pores of the nanosponges, and its availability is prolonged.

### 3.3. Tricine-SDS-PAGE of the Nisin-Z Free and Loaded with Nanosponges

**Figure 3:**
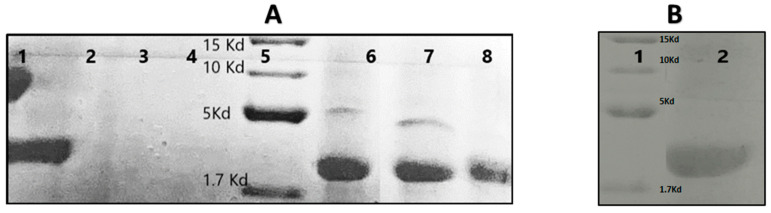
Tricine-SDS-PAGE analysis of Nisin with different treatments. (**A**) Lane 1: Nisin (4 mg/mL); Lane 2: Nisin (4 mg/mL) and pepsin (7.5 mg/mL); Lane 3: PMDA-NSs (15 mg/mL); Lane 4: CDI-NSs (15 mg/mL); Lane 5: Marker; Lane 6: Nisin and PMDA-NS; Lane 7: Nisin and CDI-NSs; Lane 8; Nisin, CDI-NSs and pepsin (7.5 mg/mL). (**B**) Lane 1: Marker; Lane 2: Nisin, PMDA-NSs and pepsin (7.5 mg/mL). The same protein quantity was loaded.

The Nisin activity is affected by protein degradation enzymes in the environments such as the digestive system. The stability and protective effects of Nisin by combination with PMDA/CDI-NSs were evaluated in the presence of pepsin, the principal acid protease of the stomach, through the Tricine-SDS-PAGE gel electrophoresis, the results of which are shown in Figure 3A. Nisin has a relative molecular mass of 3.5 kDa [43], which is located in lane 1. Meanwhile, PMDA-NSs (lane 3) and CDI-NSs (lane 4) showed no characteristic bands. The results exhibited the band of Nisin exposed to pepsin was disappeared, while when Nisin was encapsulated inside the CD-NSs in the presence of pepsin could show the bands, which might be attributed to the protective effect of PMDA/CDI-NDSs, which prevent degradation. Even a second band appeared, maybe caused by any conformational change or the complexation generated a running delay in a proportion of Nisin, creating two bands [44,45]. 

### 3.4. Intracellular Uptake of Free Coumarin-6 (C-6) in Comparison Coumarin-6-Loaded PMDA-NSs and CDI-NSs

**Figure 4:**
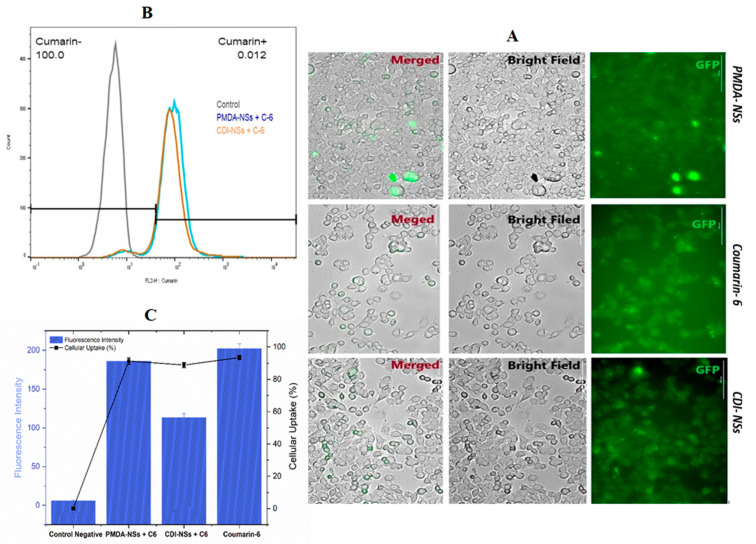
Uptake of coumarin-6 labeled CDNSs on HT-29 cells at 37 °C for 4 h was observed and investigated by fluorescent microscopy and flowcytometry. (**A**) was observed by fluorescence microscope. (**B**): Flowcytometry assay, (**C**): Mean of Fluorescence Intensity of HT-29 cells by flowcytometry assay. Data are expressed as mean ± SD (*n* = 3). One-way ANOVA analyses was used.

To evaluate the possible intracellular uptake of Nisin when complexed by PMDA-NSs and CDI-NSs, coumarin-6 (0.1 mg/mL), a fluorescence biomolecule, was used as template to simulate the uptake of Nisin. Coumarin-6 was loaded into the fixed concentration of nanosponges (10 mg/mL) for 4 h. Then, their qualitatively and quantitatively uptakes were analyzed by Cytation-5 Cell Imaging Multi-Mode Reader and Fluorescence-activated Cell Sorting (FACS), respectively, in two different cancer cells (HT-29 and MCF-7). Fluorescence images approved that coumarin-6-loaded PMDA-NSs and CDI-NSs was taken up in both HT-29 and MCF-7 cells (Figure 4A), while the HT-29 showed higher up-take (Figure 4A). However, coumarin-6-loaded PMDA-NSs was highly taken up in both cells compared with CDI-NSs. Perhaps, CD-NSs present higher complexation strength for Coumarin-6 than PMDA. To support these results, we used the quantitative method (FACS).

**Figure 5:**
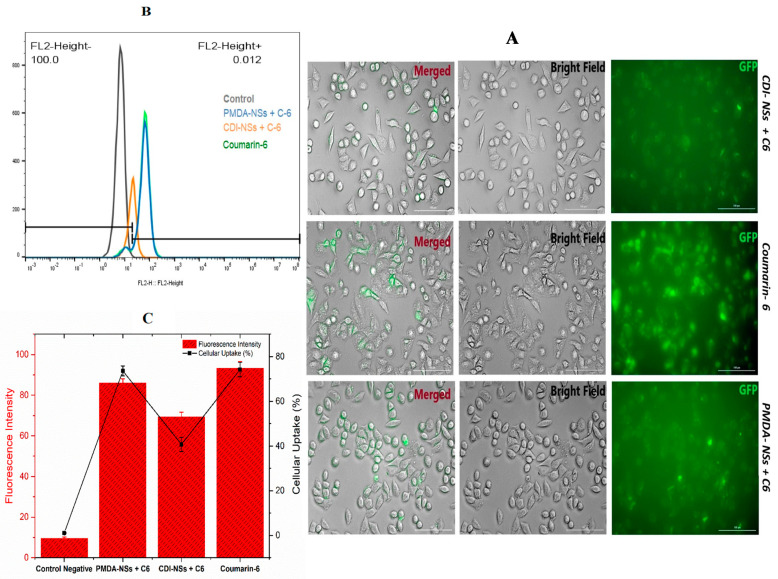
Uptake of coumarin-6 labeled CDNSs on MCF-7 cells at 37 °C for 4 h was observed and investigated by fluorescent microscopy and flowcytometry. (**A**) was observed by fluorescence microscope; (**B**,**C**) were analysis of flowcytometry data; (**B**): Flowcytometry assay, (**C**): Mean of Fluorescence Intensity of MCF-7 cells by flowcytometry assay. Data are expressed as mean ± SD (*n* = 3). One-way ANOVA analyses was used.

As shown in Figure 4C and Figure 5C, the cellular uptake percentage of C-6 complexed in PMDA-NSs was notably greater than CDI- NSs in MCF-7 cell line (*p* <0.0001), whereas in the HT-29 cell, there was not seen differences, furthermore, the mean of fluorescence intensity (MFI) of PMDA-NSs compared to CDI-NSs (*p* < 0.0001) in both cell lines was considerably higher. Surprisingly, a comparative analysis indicated the MFI and cellular uptake percentage of both CDNSs in HT-29 cells were significantly higher than MCF-7 (*p* < 0.0001), which was in agreement with microscopic fluorescence results (Figure 5). These results suggest a better uptake of selected biomolecules inside the cancer cells to release targeted drugs when they are complexed [30].

### 3.5. MTT (3-(4, 5-dimethylthiazol-2-yl) 2, 5-diphenyl Tetrazolium Bromide) Assay 

**Figure 6:**
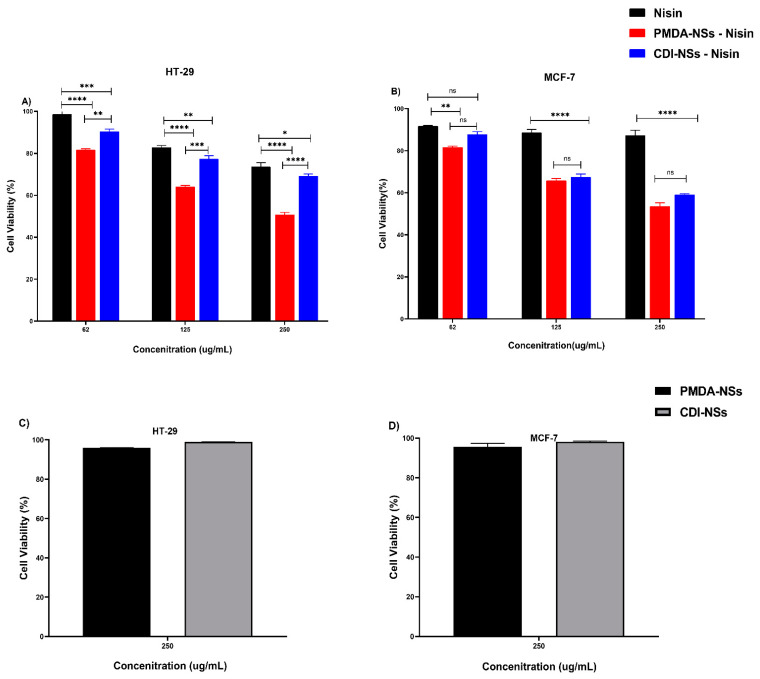
Cell viability results for colon cancer (HT-29) and breast cancer (MCF-7) cells exposed to Nisin-Z and loaded on nanosponges (**A**,**B**) and plain nanosponges (**C**,**D**) for 24 h. Results are expressed relative to the untreated control, which was set as 100% viable. Data are expressed as mean ± SD (*n* = 3). Two-way ANOVA analyses was used. * *p* < 0.05, ** *p* < 0.01, *** *p* < 0.001, **** *p* < 0.0001, and ns (Not Significant).

The cell viability was evaluated by the MTT assay. Firstly, the cytotoxic effect of blank CDI-NSs and PMDA-NSs was checked in the two cell lines (Figure 6C,D). Nanoparticles did not show cytotoxicity even at a high amount of CDNSs (250 µg/mL) toward the cells at 24 h, indicating that selected nanoparticles are nontoxic to tumor cells at selected concentrations. Nisin Z free and loaded on both nanoparticles showed a dose-dependent cytotoxicity to both of the tumor cells. As shown in Figure 6B, the cytotoxicity of free-Nisin was not significant against MCF-7 cells, but interestingly, Nisin loaded on both NSs showed the viability of cells was considerably lower in all concentrations compared to Nisin (*p* < 0.0001), while there was not seen the differences cytotoxicity effect between Nisin encapsulated with PMDA-NS and CDI-NSs. On the other hand, free Nisin indicated a good cytotoxicity effect against colon cancer cell in comparison with breast cancer, which might be associated to differences of the Nisin anti-cancer mechanism effect in these cells (Figure 6A,B). Furthermore, Nisin loaded on PMDA-NSs illustrated greater cellular toxicity than free Nisin and encapsulated with CDI-NSs even at 250 µg/mL Nisin loaded on PMDA-NSs displayed cytotoxicity about two folds higher than that of free-Nisin after 24 (*p* < 0.0001). However, it seems due to the lack of minimum dosage that is required to obtain the significant toxicity, both Nisin and Nisin loaded on CDI-NSs exhibited similar toxicity to the MCF-7 cells at the low concentration. This result suggested that the enhanced cytotoxicity of Nisin loaded on PMDA-NSs in both cells might be due to the higher bioavailability of PMDA-NSs which increased the intracellular concentration of Nisin in both cells, in particular HT-29 [30].

### 3.6. LDH Release

**Figure 7:**
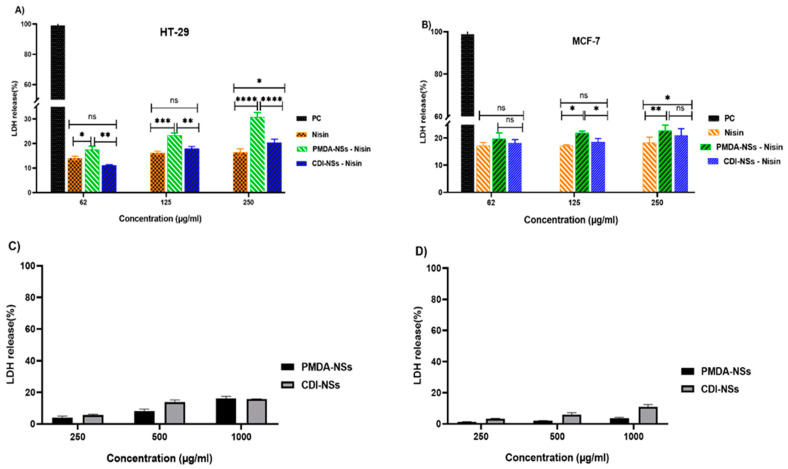
LDH release from HT-29 and MCF-7 cells following treatment with free Nisin Z and loaded on nanosponges (**A**,**B**) and plain nanosponges (**C**,**D**) for 24 h. Results are expressed relative to the untreated control and the maximum release sample (Positive Control (PC)), which was set as having 0 and 100% LDH release, respectively. Data are expressed as mean ± SD (*n* = 3). Two-way ANOVA analyses was used. * *p* < 0.05, ** *p* < 0.01, *** *p* < 0.001, and **** *p* < 0.0001 and ns (Not Significant).

Lactate dehydrogenase (LDH) was used to determine whether the observed cytotoxicity of free Nisin Z and loaded on nanosponges against both cancer cells was related to cell membrane damage. The LDH method, which measures the release of cytosolic enzyme lactate dehydrogenase (LDH) in culture media after cell plasma membrane damage, is theoretically directly comparable to the number of lysed cells. Firstly, the release of LDH in two cancer cells, which treated with the blank CDI-NSs and PMDA-NSs, was evaluated (Figure 7C,D). Nanoparticles did not show notable amount of LDH release even at a high concentration of CDNSs (1000 µg/mL) against cancer cells at 24 h, suggested that our nanoparticles are not able to damage the cell membrane of tumor cells. Interestingly, Nisin free and loaded on both nanoparticles was dose-dependent to stimulate tumor cells to release the LDH. This assay illustrated that the colon cancer cells treated by free Nisin resulted in a considerably larger increase in LDH release than MCF-7 cells, indicating membrane damage, while Nisin encapsulated by CDI-NSs except in last concentration did not show differences effect on LDH release compared to Nisin free in both cells (Figure 7A,B). On the other side, colon cancer cells exposed to Nisin loaded on PMDA-NSs resulted in a dramatic increase in LDH release about two times more at 250 µg/mL compared to free Nisin (*p* < 0.0001), also this formulation showed more increment in LDH release in MCF-7 cells at the last concentration than free Nisin.

### 3.7. Detection of Apoptosis and Late Apoptosis/Necrosis with Flow Cytometry

**Figure 8:**
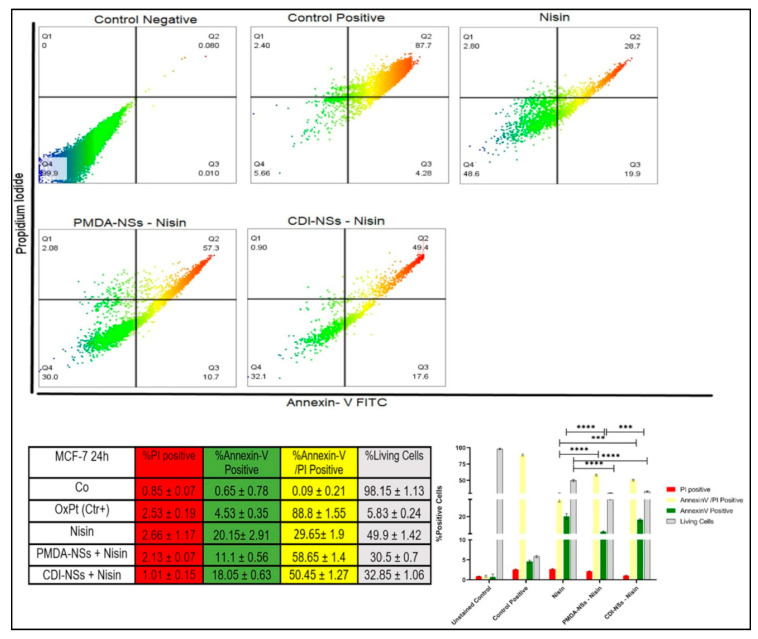
Representative flow cytometric dot-plots indicating the population sizes of apoptotic and necrotic in MCF-7 cells after exposure to 250 µg/mL of Nisin Z and loaded on PMDA/CDI-NSs for 24 h. Analyses were performed with annexin-V-FITC (FITC) and propidium iodide (PI). The untreated control was not exposed to Nisin Z and the complexes, and positive control was treated with oxaliplatin (OxPt). Experiments were done in triplicate and independently repeated. Bar graphs illustrate the percentage of apoptotic, necrotic, and late-stage apoptotic MCF-7 cells after exposure to free Nisin Z and encapsulated by the NSs. *** *p* < 0.001, **** *p* < 0.0001 apoptosis relative to the free Nisin Z. Data are expressed as mean ± SD (*n* = 3). Two-way ANOVA analyses was used.

**Figure 9:**
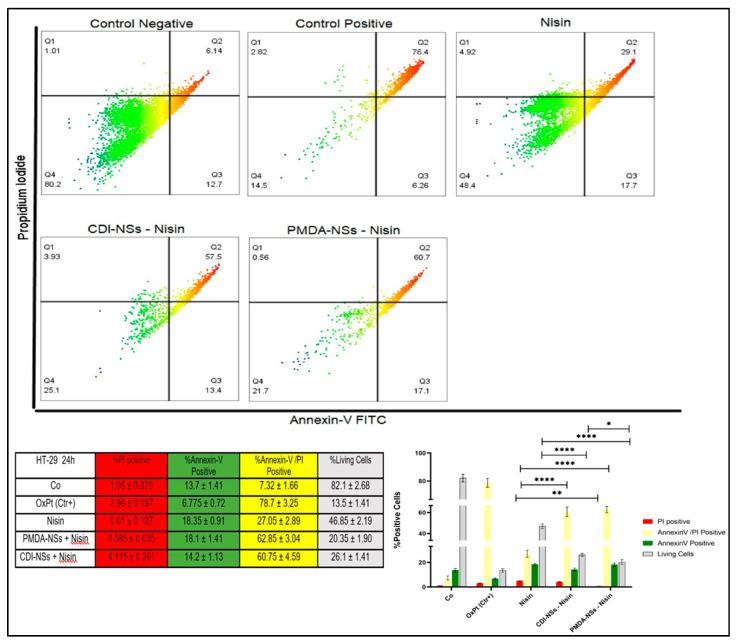
Representative flow cytometric dot-plots indicating the population sizes of apoptotic and necrotic in HT-29 cells after exposure to 250 µg/mL of Nisin Z and loaded on PMDA/CDI-NSs for 24 h. Analyses were performed with annexin-V-FITC (FITC) and propidium iodide (PI). The untreated control was not exposed to Nisin Z and the complexes, positive control was treated with oxaliplatin (OxPt). Experiments were done in triplicate and independently repeated. Bar graphs illustrate the percentage of apoptotic, necrotic, and late-stage apoptotic HT-29 cells after exposure to free Nisin Z and encapsulated by the NSs. * *p* < 0.05, ** *p* < 0.001, **** *p* < 0.0001. Data are expressed as mean ± SD (*n* = 3). Two-way ANOVA analyses was used.

Cytotoxicity and LDH release assays showed a concentration-dependent decrease in cell viability and an increase in LDH release among cancer cells treated with free Nisin Z and encapsulated by nanosponges. The assays also suggested that free and loaded Nisin Z on PMDA-NSs appeared to be more selectively toxic to colon cancer cell line, while Nisin loaded to CDI-NS showed less toxic effect on the breast cancer cells. Therefore, we implied the flow cytometry to understand that if the observed lower cell viability in cancer cells was of necrotic or apoptotic origin. Cultured colon and breast cancer cells were exposed to the same concentration of free Nisin Z (250 µg/mL) and CDNSs suspension containing the same amount of entrapped Nisin Z based on the results of the cytotoxicity assays and LDH release, the FITC AnnexinV flow cytometry assay was used for analysis. The results of MCF-7 cells treated with free Nisin Z indicated that with approximately 30% of the cells undergoing late apoptosis/necrosis while the Nisin/PMDA-NSs complex showed notable increase about two times more than free Nisin (*p* < 0.0001). Interestingly, breast cancer cells exposed to free Nisin and Nisin/CDI-NSs presenting early apoptotic markers of about 20%, which was two times higher to Nisin/PMDA-NSs. In addition, the % of cell viable in Nisin entrapped by both NSs was markedly less than Nisin (*p* < 0.0001) (Figure 8). On the other hand, the percentage of live cells in HT-29 cancer cells was dramatically reduced to 20.35 and 26.1% for Nisin/PMDA-NSs and Nisin/CDI-NSs, respectively, which was two times more than free Nisin (*p* < 0.0001). These results suggest that death of HT-29 cancer cells exposed to Nisin loaded on both NS is likely due to activation of an apoptotic pathway (late apoptosis/necrosis), while MCF-7 cancer cells underwent early apoptotic pathway (Figure 9). The Nisin-Z /PMDA-NSs induced far better apoptosis in both cancer cells compared to Nisin free and Nisin/CDI-NSs. These outcomes substantiate the cytotoxicity data of MTT assay and LDH release that showed free Nisin Z and loaded on PMDA/CDI-NSs are more selectively toxic towards HT-29 cells than MCF-7 cancer cells. 

## 4. Discussion

Peptides and proteins with biological activities nowadays have been introduced as therapeutic (particularly anti-cancer) agents to kill the cancer cells, while their limitations including difficulty for transportation through the cell membrane and enzymatic degradation induced by either the reticuloendothelial or digestive system has remained [46]. Effective delivery of anti-cancer drugs in connection with nanoparticles is a new window in cancer treatment. The specific structure of β-CD-NS led to their consideration as a prominent carrier [30]. Particularly, in addition to the biocompatibility typical of polysaccharides, they can interact with biological tissues via their tunable functional groups, which makes CD-NSs a good tool for targeted drug delivery [33,47,48].

PMDA and CDI nanosponges showed encapsulation efficiency of 91 and 92% for Nisin Z, respectively, but also they revealed significant drug loading capacity, as previous studies reported the high encapsulation efficiency of CD-NSs for various drugs [33,49,50]. This result was in accordance with the outcomes of other studies that revealed the high encapsulation efficiency of resveratrol, oxyresveratrol, bovine serum albumin (BSA), and insulin complexed by different nanosponges [42,51,52]. The release profile and pepsin degradation indicates that CD-NSs are able to protect the peptide of specific degradation and improve its release of the peptide. These aspects are important in digestion processes to achieve the tumor with the peptide in functional conditions [40]. It has been suggested that for insulin, as a peptide case, stability can be ameliorated in the presence of hydroxylpropyl-β-cyclodextrin (HP-β-CD) and cationic cyclodextrin polymers (CPβCDs) in different environments such as in simulated intestinal fluid, which was mainly because of retaining in the core of the nanoparticles and well protected against degradation in simulated gastric fluid [53,54]. As a case of CDNSs to protect peptides, there has been shown insulin complexed by crosslinking β-cyclodextrins with pyromellitic dianhydride increases the intestinal permeation when loaded inside the nanosponge and in-vivo studies confirmed the presence of insulin in rat plasma and a marked hypoglycemic effect in diabetic mice after oral administrations [42]. The cellular uptake outcomes illustrated the Coumarin-6 were internalized into the cancer cells effectively, suggesting the uptake of Nisin too. Previous studies indicated that the nanosponges as drug carriers can effectively deliver the biological agents inside the cancer cells [33,55]. The results of MTT and LDH assay illustrated that the cell cytotoxicity and membrane damage in both HT-29 and MCF-7 cancer cells depended on concentration of free Nisin Z and encapsulated with NSs, in particular, Nisin loaded on PMDA-NSs showed better anti-cancer effect against colon (HT-29) cancer that was concordant with other studies outcomes that showed a significant reduction of cell viability in colon [56], melanoma [3], and MCF-7 [11] cancer cells when they were exposed to Nisin-Z, whereas in this study the Nisin Z cytotoxicity effect was remarkably enhanced in complexed with NSs against both cancer cells, which might be related to better entrance of nanosponges into the cells to release Nisin inside the cancer cells. 

It has been suggested that the net positive charge of Nisin Z with anionic cell membrane of the cancer cells preferentially interact with phosphatidylcholine, leading to a better transmembrane potential than normal cell membranes because of more cholesterol content in normal cells [9,14,57]. One of the main mechanisms of cancer cells is to strongly suppress the apoptosis (programmed cell death), which may lead to cancer chemotherapy resistance and uncontrolled condition [58], as previous studies revealed Nisin plays its role in apoptosis by increasing mitochondrial intrinsic pathway of apoptosis to either up regulate pro-apoptotic molecules or down regulate anti-apoptotic molecules [58,59,60,61]. In addition, Hosseini et al.’s study showed Nisin could suppress proliferation of SW48, HT29, and Caco2 cells by regulation of the metastatic genes in colorectal cancer cells [56] as well as Ahamdi et al.’s study showed that the bax / bcl-2 ratio in the colon adenocarcinoma cell line raised during treatment with Nisin due to activation of the apoptotic pathway [62]. So, implying Nisin as an inducer of apoptosis agent maybe could control the uncontrolled condition in cancer cells, in agreement with these studies, the results of apoptosis assay in present assay showed the percentage of live HT-29 and MCF-7 cancer cells when exposed to free Nisin Z was considerably higher compared to loaded on selected NSs in the way that Nisin encapsulated with NSs moved the colon cancer cells mainly to the late apoptosis/necrosis stage. Interestingly, breast cancer cells exposed to free Nisin Z and Nisin/CDI-NSs present early apoptotic markers. The results of Preet et al.’s study emphasized the therapeutic efficacy of gold nanoparticle-assisted co-delivery of Nisin, doxorubicin, and Nisin-doxorubicin combination against murine skin cancer, probably through ROS mediated apoptotic effects and immunomodulation either alone or synergistically [4]. In another study, Goudarzi et al. evaluated the cytotoxicity effects of Nisin and Nisin-loaded PLA-PEG-PLA nanoparticles on gastrointestinal (AGS and KYSE-30), hepatic (HepG2), and blood (K562) cancer cells, the results of which showed that nanoparticles loaded with Nisin increased the percentage of apoptotic cells and the effect of cytotoxicity on the mentioned cancer cell lines more than free Nisin. [12].

## 5. Conclusions

Based on the results of this study, we can conclude that PMDA and CDI-NSs polymeric particles are able to carry Nisin Z, a peptide drug. This non-toxic delivery system showed notable uptake into MCF-7 and HT-29 cell lines, and the higher toxicity and cell membrane damage were shown by Nisin-Z encapsulated with NSs as compared to that of free Nisin Z against both cancer cells in MTT and LDH assays. Furthermore, Nisin Z free and loaded on PMDA/CDI-NSs are more effective towards HT-29 cells than MCF-7 cancer cells and indicates that the cell death observed in these cells is most likely due to the activation of an apoptotic pathway. Finally, the pepsin degradation study and release profile showed that the stability of Nisin-Z in presence of CD-NSs complexes was increased, which suggests the complexes would be interesting to deliver protein drugs, which have low stability in the gastrointestinal tract, suggesting a promising future application.

## Figures and Tables

**Table 1:** Particle size and Zeta potential of drug and drug-loaded nanosponges (error reported as SMD).

Carriers	Z-Average (nm)	Zeta Potential (mV)	PDI	EE (%)	DL (%)
CDI-NSs	164.3 ± 1.2	−16.5 ± 0.3	0.22 ± 0.04	-	-
CDI-NSs + Nisin	187.8 ± 2.4	−13.8 ± 0.3	0.41 ± 0.07	91	22.74
PMDA-NSs	308 ± 0.9	−20 ± 0.5	0.41 ± 0.02	-	-
PMDA-NSs + Nisin	369 ± 3.6	−14.0 ± 0.6	0.54 ± 0.09	92	23.3

## Data Availability

Not applicable.
